# Physicochemical Characterization of Fucoidans from *Sargassum henslowianum* C.Agardh and Their Antithrombotic Activity In Vitro

**DOI:** 10.3390/md20050300

**Published:** 2022-04-28

**Authors:** Peichun Lin, Suhua Chen, Min Liao, Weimin Wang

**Affiliations:** 1 School of Chemistry and Environment, Guangdong Ocean University, Zhanjiang 524088, China; 2111911013@stu.gdou.edu.cn; 2 School of Food Science and Technology, Guangdong Ocean University, Zhanjiang 524088, China; moxiaowei11@stu.gdou.edu.cn (M.L.); wwaa1816@163.com (W.W.)

**Keywords:** *Sargassum henslowianum* C.Agardh, fucoidan, physicochemical characterization, HUVEC, antithrombotic

## Abstract

Sargassum fucoidan is a kind of sulfated heteropolysaccharide with a variety of biological activities. The aim of this study was to investigate the extraction, purification, physicochemical characterization and in vitro antithrombotic activity of fucoidan from *Sargassum henslowianum* C.Agardh. Hot-water-assisted ultrasound was used to extract fucoidan (F). Fucoidan was purified by DEAE cellulose 52 (F1), Vc-H_2_O_2_ (FD1) and Superdex 75 gel (FDS1). The physical and chemical properties of fucoidans were analyzed by chemical composition, monosaccharide composition, average molecular weight (Mw) and FTIR. The sulfate contents of F, F1, FD1 and FDS1 were 11.45%, 16.35% and 17.52%, 9.66%, respectively; the Mw was 5.677 × 10^5^, 4.393 × 10^5^, 2.176 × 10^4^ and 6.166 × 10^3^, respectively. The results of monosaccharide composition showed that the four fucoidans contained l-fucose, d-galactose, l-mannose, d-xylose, l-rhamnose and d-glucose, but the mass fraction ratio was different. The results of FTIR showed that fucoidan contained characteristic peaks of sugar and sulfate. In vitro, F1, FD1 and FDS1 could alleviate HUVEC damage induced by adrenaline (Adr). F1, FD1 and FDS1 decreased vWF and TF and increased the ratio of t-PA/PAI-1 in Adr-induced HUVEC.

## 1. Introduction

Seaweed is a potential renewable resource in the marine environment and plays an important ecological role in the ocean [1]. Seaweed has a variety of active components and is a promising new type of biochemical active substance [2]. *Sargassum* is a kind of brown algae, which contains a variety of bioactive metabolites such as polyphenols, polysaccharides, pigments, steroids and terpenoids [3]. *Sargassum henslowianum* is an edible brown alga widely distributed in southeastern China and Southeast Asia. Previous studies have shown that *Sargassum henslowianum* is rich in polysaccharides, and the study of polysaccharides was mainly focused on the structural analysis and biological activity evaluation of fucoidan [4]. The main component of *Sargassum* polysaccharides is fucoidan.

Fucoidan is mainly composed of l-fucose and sulfate groups with a complex heterostructure. In addition, it also contains uronic acid, galactose, rhamnose and other ingredients [5]. Fucoidan from brown algae had been proven to have a variety of biological activities, such as anti-tumor [6], anti-virus [7], anticoagulant [8], antithrombotic [9] and cholesterol-lowering [10]. A wide range of biological activities showed that fucoidan is likely to become an important substance in the food, pharmaceutical and cosmetic industries in the future. The excellent biological activity of fucoidan may be that it contains different numbers of sulfate groups, and the position of sulfated groups along the macromolecular skeleton also plays an important role in its functional properties [11]. The detailed chemical structure of sulfated polysaccharides varies from species to species, as well as extraction and purification methods, harvest time and location of algae [12]. Therefore, each new purified fucoidan extracted from seaweed is a new compound with unique properties and structure and with potential biological activity.

Cardiovascular disease is the leading cause of human death all over the world, especially thrombosis [13]. Thrombus is a clot or deposit formed by blood components in flowing blood on the surface of blood vessels or cardiac intima and fibrin, which can occur in any part of the blood vessels. The factors of thrombosis often influence each other and cooperate to promote thrombosis, which can be divided into three major factors: vascular endothelial injury, abnormal blood flow and abnormal blood composition [14]. Endothelial cells are arranged on the inner surface of blood vessels, including arteries, veins and capillaries. They maintain the balance of coagulation and anticoagulation by releasing factors or providing necessary receptors. Endothelial cells are important regulators of blood coagulation [15,16]. Therefore, in this study, Human Umbilical Vein Endothelial Cells (HUVEC) were selected to study the preliminary antithrombotic effect of fucoidan. Under physiological conditions, endothelial cells form a non-adhesive surface that prevents platelet activation and coagulation cascades [17]. Once the vascular endothelium is damaged and exfoliated, the exposed subendothelial tissue (collagen) and the platelets in contact with it will be activated, resulting in thrombosis [18]. The damage of endothelial cells leads to the activation of coagulation factors, which dominates the coagulation mechanism in the body and leads to thrombosis [19]. Endothelial damage is the main manifestation of vascular wall injury. Protecting endothelium and giving full play to the antithrombotic effect of endothelial cells are of great significance to the prevention and treatment of thrombosis and thromboembolic disease. Heparin as an anticoagulant and antithrombotic agent has important clinical significance. However, because of the risk of bleeding and animal pathogen contamination, it is urgent to find and develop new natural antithrombotic drugs with good safety and little side effects [20].

In this study, crude fucoidan from *Sargassum henslowianum* C.Agardh was extracted by hot-water-assisted ultrasound and was purified by DEAE cellulose 52, Vc-H_2_O_2_ and Superdex 75 gel column. The physicochemical characterization of fucoidans was studied by chemical analysis, Fourier transform infrared spectroscopy (FTIR), average molecular weight (Mw) and monosaccharide composition. Furthermore, the antithrombotic activity of fucoidans was evaluated by in vitro experiments.

## 2. Results

### 2.1. Isolation and Purification of Fucoidans

**Figure 1:**
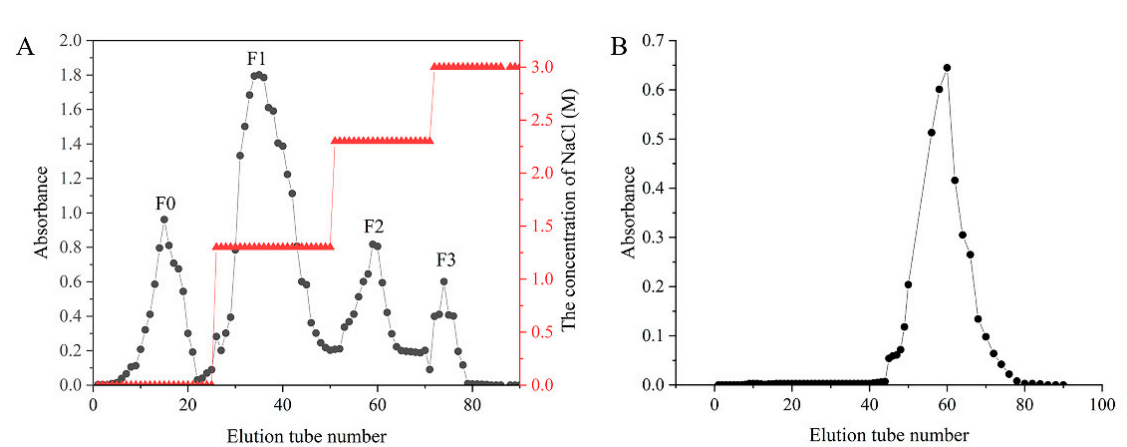
Stepwise elution curve of F and FD1. (**A**) Stepwise elution curve of F on the DEAE cellulose 52 ion column chromatography column. The fucoidans obtained from 0, 1.3, 2.3 and 3.0 M NaCl eluents were called F0, F1, F2 and F3, respectively. (**B**) Stepwise elution curve of FD1 on the Superdex 75 gel column chromatography column.

Using *Sargassum henslowianum* as raw material, crude sargassum fucoidan F was obtained by ultrasonic hot water extraction, and the yield was 6.25%. Four fucoidans, F0, F1, F2 and F3, were obtained from F purified by DEAE cellulose 52 ion column using different concentrations of NaCl solution as eluent phase as shown in Figure 1A. According to the peak area, the content of F1 was the highest, and the yield was 65.2%. With the increase in eluent concentration, the total sugar content of eluted components decreased gradually. Therefore, F1 was selected to degrade using Vc-H_2_O_2_. As shown in Figure 1B, FD11 was obtained by eluting the degradation product FD1 with the Superdex 75 gel column.

### 2.2. Chemical Composition, Mw and Monosaccharide Composition of Fucoidans

As shown in Table 1, the chemical composition of the four fucoidans was different. The total sugar content of F, F1 and FD1 was 27.97%, 35.14% and 49.27%, respectively. The sulfate content of F, F1 and FD1 was 11.45%, 16.35% and 17.52%, respectively. The total sugar content and sulfate content of FD1 were higher than those of F and F1. The uronic acid content of F, F1 and FD1 was 14.47%, 12.54% and 10.85%, respectively. Because of the small amount of FDS1 extracted, we only determined the content of sulfate. The sulfate content of FDS1 was 9.66%.

The Mw of F, F1, FD1 and FDS1 was 5.677 × 10^5^, 4.393 × 10^5^, 2.176 × 10^4^ and 6.166 × 10^3^, respectively. The Mw of FDS1 purified by DEAE cellulose 52, degradation and Superdex 75 gel column was the smallest. The FD1 purified by DEAE cellulose 52 and degraded by Vc-H_2_O_2_ took second place. The Mw of F without purification was the largest. It also showed that DEAE cellulose 52, degradation and Superdex 75 gel column were effective in the purification of fucoidan.

**Figure 2:**
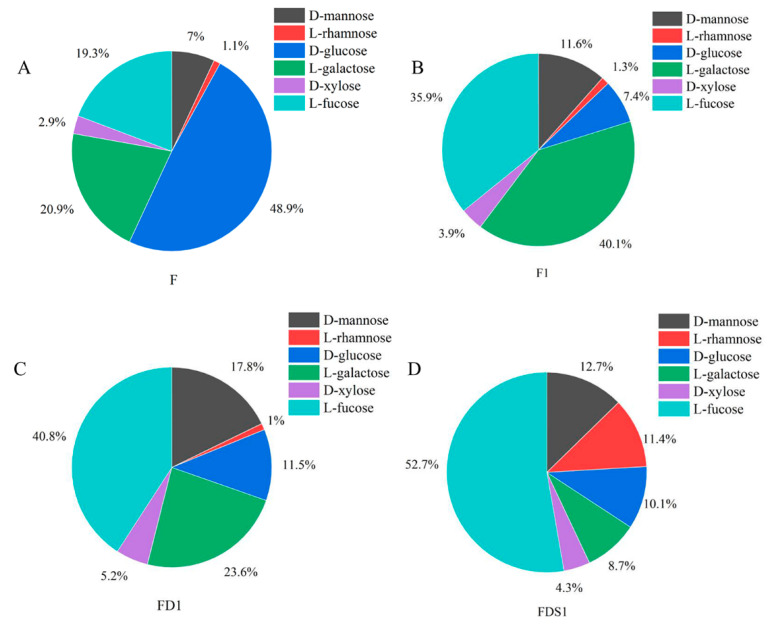
Monosaccharide composition: (**A**) F, (**B**) F1, (**C**) FD1, (**D**) FDS1.

The monosaccharide composition of the four fucoidans is shown in Figure 2. The monosaccharide composition of F, F1, FD1 and FSD1 was the same, but the mass percentage content was different. The monosaccharides of F, F1, FD1 and FSD1 include d-mannose, l-rhamnose, d-glucose, l-galactose, d-xylose and l-fucose. In the monosaccharide composition of F, d-glucose, l-galactose and l-fucose were the main parts. The content of d-glucose in F was as high as 48.9%. The mass fractions of l-galactose and l-fucose in F were 20.9% and 19.3%, respectively. The contents of d-mannose, l-rhamnose and d-xylose were all less than 10%. l-fucose and l-galactose were the main components of F1 monosaccharides, accounting for 35.9% and 40.1%, respectively. The contents of d-mannose and d-glucose were only 11.6% and 7.4%. The contents of d-mannose and d-glucose were 11.6% and 7.4%. In the monosaccharide composition of FD1 and FDS1, the content of l-fucose was the highest, which was 40.8% and 52.7%, respectively. The contents of l-galactose (23.6%) and d-mannose (17.8%) were also higher in FD1. In FDS1, the content of l-fucose was more than half, followed by d-mannose and l-rhamnose, accounting for 12.7% and 11.4%. d-xylose was present in lesser amounts in the four fucoidans. We found that the content of l-fucose in fucoidan increased with the purification of fucoidan.

### 2.3. FTIR Spectrum of Fucoidans

**Figure 3:**
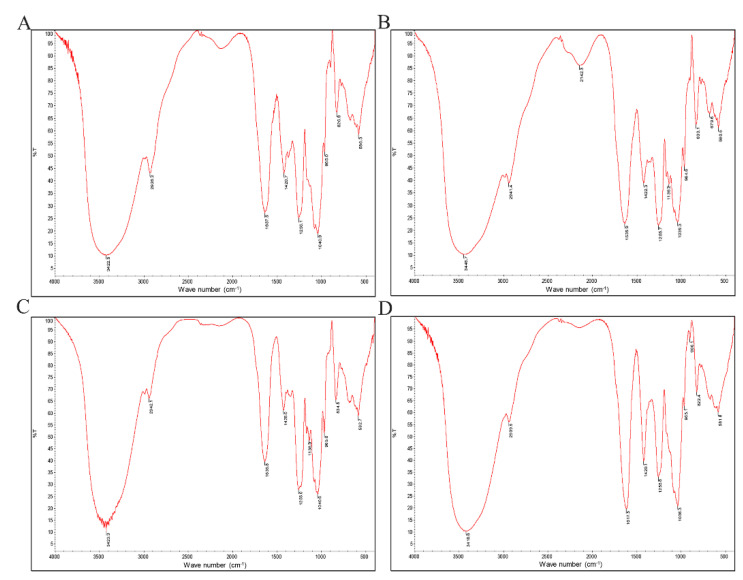
FTIR spectrum of sulfated fucoidans: (**A**) F, (**B**) F1, (**C**) FD1, (**D**) FDS1.

The FTIR spectrum of the four fucoidans was basically the same. As shown in Figure 3, there was a broad absorption peak caused by the stretching vibration of intramolecular or intermolecular hydrogen bonds of polysaccharides at 3418–3500 cm^−1^. The results showed that the extracted fucoidan had C-H stretching vibration at 2928–2942 cm^−1^ and carbohydrate C-H deformation vibration from 1420–1425 cm^−1^. There was an amide group C=O stretching vibration peak at 1617–1637 cm^−1^. There was a stretching vibration peak of S=O at 1255 cm^−1^, indicating that it contained sulfate radicals. The absorption peak was caused by the stretching vibration of C-O-C, by the angular vibration of C-O on the sugar ring C-O-C at 1050 cm^−1^, and by the characteristic absorption peak of β-glycosidic bond at about 897 cm^−1^.

### 2.4. Cell Viability

**Figure 4:**
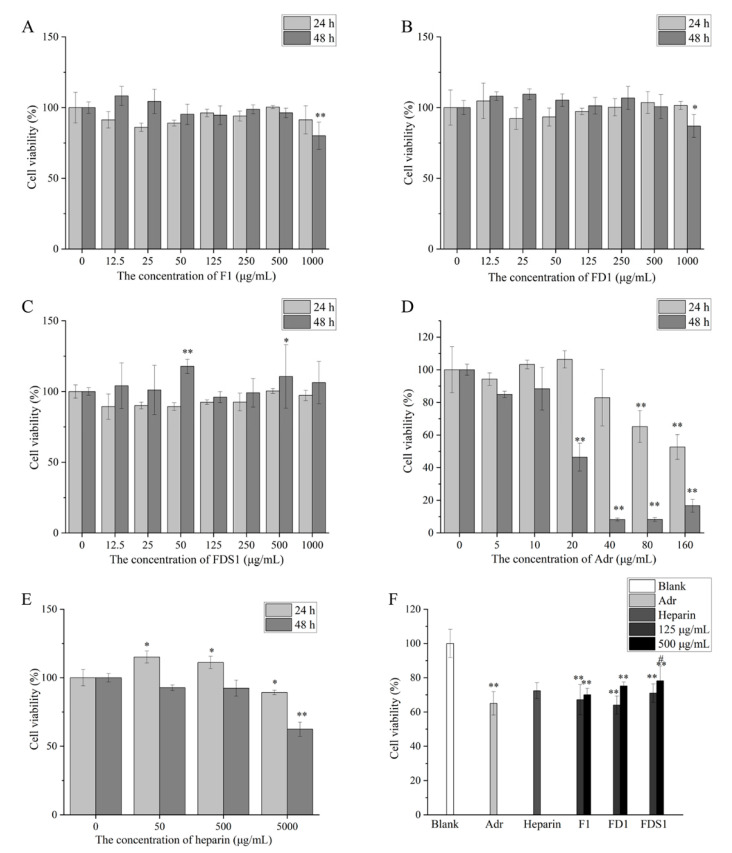
Cell viability: (**A**) F1, (**B**) FD1, (**C**) FDS1, (**D**) Adr, (**E**) Heparin, (**F**) effects of fucoidan on Adr-induced HUVEC cell viability. Compared with the concentration of fucoidan 0 or Blank, the significance is expressed by * *p* < 0.05 and ** *p* < 0.01. Compared with the Adr group, the significant difference is expressed by # *p* < 0.05. Blank means that HUVEC only exposes DMEM medium. Adr refers to the negative control. Heparin means the positive control.

As shown in Figure 4B,C, the concentrations of F1, FD1 and FDS1 used in this experiment are not toxic to HUVEC within 24 h. However, after 48 h of exposure, high concentrations of F1 and FD1 (1000 μg/mL) significantly decreased cell viability of HUVEC. Other concentrations of fucoidan did not affect cell viability of HUVEC, and even 50 and 500 μg/mL FDS1 significantly promoted the viability of HUVEC. F1, FD1 and FDS1 with concentrations of 125 and 500 μg/mL were selected to perform the follow-up experiment. As shown in Figure 4D, 5–40 μg/mL Adr had no effect on cell viability, while 80 μg/mL Adr significantly reduced cell viability by 65.15%. Furthermore, 160 μg/mL Adr resulted in only 52.65% cell viability of HUVEC. When the exposure time was 48 h, 80 μg/mL Adr significantly reduced cell viability, and cell viability was only 8.15%. Therefore, Adr with a concentration of 80 μg/mL and exposure time of 24 h was selected as the negative control group. Figure 4E showed that 50 and 500 μg/mL heparin was not toxic to cells at 24 and 48 h, while 5000 μg/mL heparin significantly inhibited cell viability. Furthermore, 500 μg/mL heparin was selected for subsequent experiments. As shown in Figure 4E, fucoidan (F1, FD1 and FDS1) can inhibit Adr-induced HUVEC damage. Compared with control, 500 μg/mL FD1 and FDS1 significantly increased cell viability. Low concentrations of F1, FD1 and FDS1 inhibited Adr-induced cell injury, but this was not statistically significant. High concentrations of FD1 and FDS1 attenuate Adr-induced HUVEC injury better than heparin, but not significantly.

### 2.5. F1, FD1 and FDS1 Affected the Levels of Cytokines Secreted by Adr-Induced HUVEC

**Figure 5:**
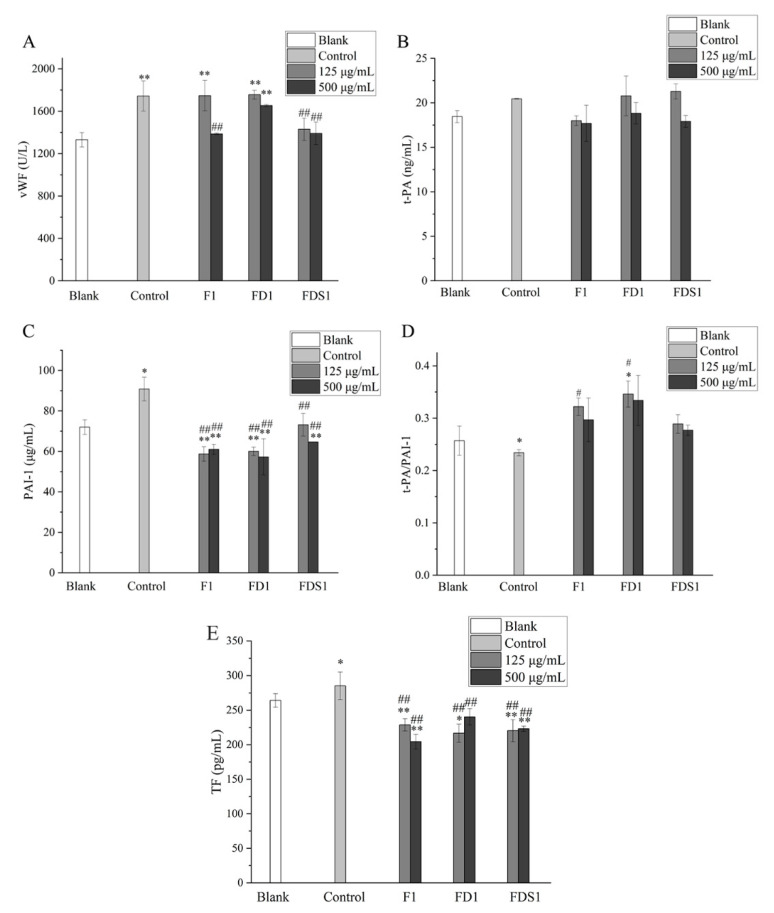
F1, FD1 and FDS1 affects the levels of cytokines secreted by Adr-induced HUVEC: (**A**) vWF, (**B**) t-PA, (**C**) PAI-1, (**D**) t-PA/PAI-1, (**E**) TF. Compared with the concentration of fucoidan 0 or Blank, the significance is expressed by * *p* < 0.05 and ** *p* < 0.01. Compared with the Adr group, the significant difference is expressed by # *p* < 0.05 and ## *p* < 0.01. Blank means that HUVEC only exposes DMEM medium. Control refers to the negative control.

As shown in Figure 5A, 125 μg/mL F1 did not reduce the secretion of vWF, while 500 μg/mL F1 significantly decreased the secretion of vWF (*p* < 0.01). FD1 can reduce the secretion of vWF, but it was not statistically significant. FDS1 significantly decreased the secretion of vWF by HUVEC (*p* < 0.01). Low concentrations of FD1 and FDS1 almost had no effect on t-PA secreted by HUVEC (Figure 5B). High concentrations of F1, FD1 and FDS1 decreased the secretion of t-PA, but there was no statistical significance. As shown in Figure 5C, fucoidan (F1, FD1, FDS1) could significantly reduce the secretion of PAI-1 by HUVEC (*p* < 0.01). t-PA and PAI-1 were a pair of antagonists, and the ratio of t-PA/PAI-1 was used to evaluate the effect of HUVEC on fibrinolysis. Compared with the control group, F1, FD1 and FDS1 can increase the ratio of t-PA/PAI-1 in Figure 5D. Finally, TF was detected and significantly reduced by F1, FD1 and FDS1 (*p* < 0.01). Heparin was used as a positive control, and the results are shown in Table 2. Compared with heparin, FDS1 can reduce the secretion of vWF by HUVEC, especially the high concentration of FDS1. The effects of F, FD1 and FDS1 on t-PA/PAI-1 and TF were similar to those of heparin. In summary, the antithrombotic effect of F, FD1 and FDS1 was similar to heparin, and the effect of a high concentration of FDS1 on reducing vWF was better than heparin.

## 3. Discussion

Fucoidan is a kind of water-soluble polysaccharide, which has potential therapeutic and drug applications because of its extensive biological activity. Marine brown algae is the main source of fucoidan. Thus, we isolated and purified fucoidan from *Sargassum henslowianum* C.Agardh and identified its structure. Ultrasonic technology is considered to be a valuable tool for extracting fucoidan. The increase in mass transfer caused by sound-induced cavitation in liquid medium is beneficial. Cavitation affects the chemical process in the system, mainly by increasing the reaction rate of existing processes or by initiating new reactions [21]. Therefore, in this study, hot-water-assisted ultrasound was used to extract crude fucoidan. Anion-exchange chromatography is a purification technology, which can be used for molecules with strong anion properties. The degradation of polysaccharides is one of the effective ways to improve its biological activity. Vc tends to promote the degradation of polysaccharides by hydrogen peroxide. Low concentrations of Vc degrade polysaccharides derived from natural products through hydroxyl radicals [22]. The separation effect of gel chromatography is good, the repeatability is high, and the separation process does not involve a change in chemical bonds; thus, it has no adverse effect on the activity of the separation. We used DEAE cellulose 52, Vc-H_2_O_2_ and Superdex 75 gel to purify crude fucoidan. After purification, the total sugar content of F1 and FD1 was 7.17% and 21.3% higher than F, and the content of the sulfate group was 4.9% and 6.07% higher, respectively. The monosaccharide composition of purified fucoidan also changed. As a monosaccharide derived from seaweed, l-fucose accounts for a large part of its composition. After many times of purification, the l-fucose content of FDS1 was the highest, followed by FD1 and F1, and the content of F l-fucose without purification was the lowest. The percentage of d-glucose in the monosaccharide composition of F was as high as 48.9%, because F is a crude fucoidan sulfate and contains a large amount of neutral glucose. After elution with DEAE cellulose 52 column, the neutral sugar was washed away by distilled water, and the mass percentage of d-glucose in the purified component F1 was reduced. Our results confirmed that DEAE cellulose 52, Vc-H_2_O_2_ degradation, and Superdex 75 gel could increase the content of l-fucose in fucoidan.

lfucose is the main monosaccharide in fucoidan, and there are other monosaccharides in fucoidan, such as xylose, mannose, galactose, glucose and rhamnose. The composition of the monosaccharide also affects the activity of fucoidan. It is generally believed that higher l-fucose content is beneficial to the extensive biological activity of fucoidan [23]. The l-fucose content of FDS1 was higher than FD1 and F1. We found that the effect of FDS1 on the recovery of Adr-induced HUVEC activity induced by Adr was better than that of FD1 and F1 (Figure 4F). Compared with F1 and FD1, FDS1 has the best effect on reducing vWF (Figure 5A). Fucoidan from *S. japonica* with high content of galactose prolonged activated partial thromboplastin time and enhanced anticoagulant activity [24]. Fucoidan with lower xylose content showed stronger anticoagulant activity [25]. Our results showed that a high concentration of F1 was better than FD1 and FDS1 in reducing TF (Figure 5E), which may be related to the highest galactose content and the lowest xylose content of F1. Zhou et al. detected the Mw of fucoidan purified by DEAE cellulose column and Sephadex G-100 gel and found that the Mw was reduced compared with crude fucoidan [26]. As expected, the Mw of FDS1 after many purification steps was the smallest, and the Mw of F without purification was the largest.

The preliminary structure of the extracted fucoidan was characterized by FTIR. The results of FTIR showed that the functional groups of F, F1, FD1 and FDS1 were almost the same, and all had the characteristic peaks of sugar and S=O. The experimental results of Shi et al. also showed that the FTIR spectrum of hydrothermal depolymerization of fucoidan was almost the same as that of untreated fucoidan [27].

Our results showed that 0–500 μg/mL fucoidan from *Sargassum henslowianum* C.Agardh did not inhibit the viability of HUVEC cells. Even 50 and 500 μg/mL FDS1 exposure for 48 h significantly enhanced cell viability. Ma et al. also found that 6.3–100 μg/mL simulated digestive product of fucoidan from *Sargassum fusiforme* could significantly increase K562 cells viability [28]. MTT assay confirmed that low molecular weight fucoidan from Sargassum horneri slightly increased HaCaT cell viability [29]. Our results were the same in that fucoidan was non-toxic to cells and even promoted cell vitality. Marine-derived sulfated l-fucose showed anticoagulant and antithrombotic effects similar to heparin. We have previously shown that fucoidan protects HMVEC damage induced by low-density lipoprotein [30]. Our previous work confirmed that sargasso fucoidan sulfate had good antithrombotic activity and less bleeding risk through in vivo experiments [31]. The same modeling agent (Adr) was used in this experiment to detect the preliminary antithrombotic activity of sargassum fucoidan in vivo. Our results indicated that fucoidan could enhance cell viability of Adr-induced HUVECs.

vWF is a large plasma glycoprotein that mediates platelet adhesion and is produced by endothelial cells. vWF is a key component that causes clots in blood [32]. Population-based studies have shown that elevated vWF levels are a risk factor for thrombosis, especially in patients with previous cardiovascular disease and in the elderly [33]. Our results suggest that F1, FD1 and FDS1 can reduce Adr-induced reduction of vWF secreted by cells, especially FDS1. The decrease in vWF level can block the binding of platelets to collagen on the injured endothelial surface, thus inhibiting platelet activation, inhibiting platelet adhesion and aggregation, and playing an antithrombotic effect. Fibrinolysis is a key step in hemostasis. The decrease in fibrinolysis is also an important risk factor for thromboembolism [34]. Endothelial cells are highly involved in this process through the expression of t-PA, urokinase type plasminogen activator (u-PA), u-PA receptor and PAI-1. t-PA released by vascular endothelial cells is the main PA in plasma. t-PA converts more plasminogen in the blood into plasmin, which can directly dissolve the thrombus [35]. PAI-1 is the main endogenous inhibitor of the fibrinolytic system [36]. Therefore, the balance between t-PA and PAI-1 can determine procoagulant and low fibrinolytic activity [37]. The increase if the t-PA/PAI-1 ratio showed thrombolytic activity. Adr led to the decrease if this ratio, and fucoidan used in the experiment could alleviate the effect and increase this ratio. TF, a cell membrane-anchored protein and a member of the cytokine receptor family, is the main activator of the coagulation system and one of the prethrombotic mediators [34,36]. Surprisingly, our results show that fucoidan can significantly reduce the amount of TF secreted by Adr-induced HUVEC.

## 4. Materials and Methods

### 4.1. Chemicals and Reagents

*Sargassum henslowianum* C.Agardh was collected from the sea around Naozhou Island, Zhanjiang City, Guangdong Province, China. The breeding of *Sargassum henslowianum* C.Agardh begins at the end of March. When the tide receded, *Sargassum* growing on the rocks was picked. It was collected in the spring of 2016 (March) and identified by Professor Xie Enyi of Guangdong Ocean University. After cleaning, the *Sargassum henslowianum* C.Agardh was air-dried under natural conditions and dried in a blast drying box at 50 °C for 1 h. After preliminary crushing, the sargassum was further treated into a smaller powder with a grinder. After 80 mesh, each bag was packed in a sealed bag (200 g) and stored in an ultra-low temperature refrigerator at −80 °C (Merit, Kaltis, USA). The sargassum powder taken out according to the amount used in the experiment was stored in a dryer at room temperature. Human umbilical vein endothelial cells (HUVEC) were purchased from BeNa culture collection (BNCC); the BNCC number of HUVEC was 337616. DEAE cellulose 52 was obtained from Guangzhou Dingguo biological co., Ltd. Heparin and seven standard monosaccharides including l-fucose, d-glucose, d-mannose, l-galactose, l-rhamnose, d-xylose and l-arabinose were purchased from Shanghai Yuanye Biotechnology Co., Ltd. Adrenaline (Adr) was obtained from Harbin Sanma Animal Pharmaceutical Co., Ltd. (Harbin, China). Fetal bovine serum (FBS), DMEM, penicillin/streptomycin and 0.25% trypsin–EDTA were purchased from Gibco (NY, USA). Superdex 75 gel column, MTT and DMSO were purchased from Sigma (LA, USA). PBS buffer solution powder was obtained from Beyotime Biotechnology (Shanghai, China). vWF, t-PA, PAI-1 and TF Elisa kits were obtained from Shanghai Yinggong Elisa Kit Co., Ltd. (Shanghai, China). All other reagents used in the experiment were of analytical grade and are commercially available.

### 4.2. Extraction of Fucoidan from Sargassum Henslowianum C.Agardh

According to our previous study, crude fucoidan was extracted [38]. According to the ratio of 1:30 (g:mL), sargassum and distilled water were added, and pH was adjusted to 6.0. The solution containing sargassum was ultrasonic (KQ-500DB, Kunshan Ultrasonic instrument Co., Ltd., Kunshan, China) at 350 W and 60 °C. After ultrasound for 45 min, the solution was extracted in a water bath at 80 °C for 3.5 h. The residue was obtained after the extract was filtered with a filter cloth, the residue was reprocessed according to the above steps, and the extract was collected after filtration. The extract was centrifuged at 5000 rpm/min for 10 min, and the supernatant was evaporated with a rotary evaporator. The obtained solution was added with absolute ethanol to remove algin, acetone to remove fat, and Sevag reagent (chloroform: *n*-butanol = 4:1) to remove impurity proteins. Finally, the solution was rotary evaporated to remove the organic solvents chloroform and *n*-butanol, dialyzed with distilled water for 48 h, and freeze-dried to obtain crude fucoidan (F) and to calculate the extraction rate of F.

### 4.3. Purification of Fucoidan from Sargassum Henslowianum C.Agardh

F was purified by DEAE cellulose 52 column (2.6 × 80 cm), followed by step elution with different concentrations of NaCl. The content of polysaccharides in the eluent was tracked and detected by phenol–sulfuric acid method [39] until complete elution. The polysaccharides were obtained after 48 h desalination, rotary evaporation concentration and vacuum freeze-drying. The polysaccharides obtained from 0, 1.3, 2.3 and 3.0 M NaCl eluents were called F0, F1, F2 and F3, respectively.

The F1 with higher content was selected for next purification. The combination of Vc and H_2_O_2_ is an effective method for the degradation of polysaccharides [40]. Regarding the method of Deng et al. [41], we made few modifications. F1 was degraded with the free-radical degradation by Vc-H_2_O_2_ to obtain the FD1. In short, H_2_O_2_ (30%, 2 mL) and Vc (0.2072 g) were added to the F solution. The mixture was reacted at 30 °C for 2 h. Then, the mixture was obtained by centrifugation. The supernatant was dialyzed, concentrated and freeze-dried to obtain FD1. Finally, FD1 was purified by Superdex 75 gel column (1.6 × 80 cm) and lyophilized to obtain FDS1.

### 4.4. Chemical Composition Analysis

Phenol–sulfuric acid method [39] was used to determine total sugar content. BaCl_2_ gel method [42] was used to determine the content of the sulfate group. Carbazole colorimetry [43] was used to determine the content of uronic acid.

### 4.5. The Average Molecular Weight (Mw) Determination

Regarding the method of *Wang* et al. [44], we made few modifications. Fucoidan (F, F1, FD1, FDS1) was mixed into 10 mg/mL solution with 0.5 mol/L NaCl, was passed through 0.22 μm filter membrane, and passed through gel column. The sample volume was 1 mL, flow rate was 0.6 mL/min, about 3 mL/tube, which was followed by phenol–sulfuric acid method, and an elution curve was drawn. The external water volume (V0) was determined by blue dextran-2000, the corresponding distribution coefficient was calculated according to the elution volume (Ve) of a series of standard glucans, the lgM-Ve/V0 standard curve was drawn, and the average relative molecular weight of each sample was calculated according to the corresponding linear regression equation. The Mw of F and F1 were determined by Sephacryl Smur300 HR gel column. The molecular weights of standard glucans were determined by Superdex 75 gel column. The molecular weights of standard glucans were 1.0 × 10^4^, 4.0 × 10^4^, 7.0 × 10^4^, 1.0 × 10^5^ and 1.1 × 10^5^ Da, respectively. The molecular weights of standard glucans were 2.0× 10^4^, 1.0 × 10^4^, 9.0 × 10^3^, 7.0 × 10^3^ and 5.0 × 10^3^ Da. The total sugar concentration was assayed using the phenol–sulfuric acid method. The two standard curves and elution curves are shown in Figure S1.

### 4.6. Monosaccharide Composition Analysis

According to the method reported by Long et al. [45], the composition of monosaccharides was determined by high performance liquid chromatography (HPLC, Agilent 1100, Santa Clara, CA, USA) with ZORBAX Eclipse column as XDB-C18 column (4.6 × 250 mm, 5 μm). Fucoidan was added with 1 mL trifluoroacetic acid and sealed immediately after being filled with nitrogen and hydrolyzed at 110 °C for 8 h. Then, it was dried with nitrogen, and 1 mL of ultra-pure water was added to obtain the polysaccharide solution. Next, 200 μL polysaccharide solution, 5 mg/mL monosaccharide standard solution and mixed monosaccharide standard solution were respectively placed into a 5 mL glass tube with plug. NaOH solution and PMP derivative reagent were added successively to react for 100 min at 70 °C water bath. After cooling to room temperature, HCl was added to neutralize, then extracted with 2 mL chloroform, and the upper water phase was filtered through a 0.22 μm water system membrane.

### 4.7. Fourier Transformed-Infrared (FTIR) Spectrometric Analysis

After weighing an appropriate amount of fucoidan, and pressing into tablets with KBr, infrared scanning was carried out in the range of 4000–400 cm^−^^1^ on the FTIR (Magna 760, Thermo Nicolet Corporation, Waltham, MA, USA), and the infrared spectral characteristics were analyzed.

### 4.8. Cell Culture

According to the method of Cao et al. [46], the HUVEC cells were cultured in DMEM medium supplemented with 10% fetal bovine serum (FBS) and 1% streptomycin/penicillin. The cells were cultured in 37 °C and 5% CO_2_ humidified incubator.

### 4.9. Cell Viability

The HUVEC cells were inoculated in 96-well plates and exposed to F1, FD1 and FDS1 for 24 and 48 h, respectively. Fucoidans were dissolved in DMEM medium. The exposure concentration of fucoidans was 0,12.5, 25, 50, 125, 250, 500, 1000 μg/mL. In addition, cell viability was measured after the cells were treated with 0, 5, 10, 20, 40, 80, 160 μg/mL Adr for 24 h. The cells were cultured in 20 μL 5 mg/mL MTT solution for 4 h. After 4 h, the supernatant was discarded, and 150 μL DMSO was added to avoid light for 10 min. The absorbance was measured at 570 nm by using enzyme-labeling instrument (BioTek, Winooski, VT, USA). According to the method of Pozharitskaya et al. [47], heparin was used as the positive control. The cells were inoculated 24 h after 96-well plate, and 50, 500, 5000 μg/mL heparin was added. MTT assay was used to detect the effect of different concentrations of heparin on HUVEC viability.

Regarding the method of Ahmad et al. [48] and Chou et al. [49], we made few modifications. The HUVEC cells were inoculated in 96-well plates and exposed to F1, FD1 and FDS1 (125, 500 μg/mL) for 24 h. DMEM without fucoidan was used as blank. The cells treated with fucoidan were stimulated by Adr (80 μg/mL) and incubated for 24 h. Only Adr solution was added to the cells as a negative control. According to the method of Pozharitskaya et al. [47], heparin (500 μg/mL) was used as a positive control. Cell viability was detected by MTT assay.

### 4.10. Enzyme-linked Immunosorbent Assay (ELISA)

The HUVEC cells were inoculated in 96-well plates and exposed to F1, FD1 and FDS1 (125 and 500 μg/mL) for 24 h. Then, the cells treated with fucoidan were stimulated by Adr (80 μg/mL) and incubated for 24 h. According to the instructions of the reagent manufacturer, the cell supernatant was collected to evaluate the production levels of von Willebrand Factor (vWF), tissue-type plasminogenactivator (t-PA), plasminogen activator inhibitor 1(PAI-1), and tissue factor (TF). According to the method of Pozharitskaya et al. [47], heparin (500 μg/mL) was used as a positive control.

### 4.11. Statistical Analysis

All experimental data had three duplicate values. The Origin2021 software was used for drawing. All data were statistically analyzed by SPSS.24 software (t test and one-way ANOVA). A probability value below 0.05 (* *p* < 0.05 or # *p* < 0.05) is considered significant and a value below 0.01 (** *p* < 0.01 or ## *p* < 0.05) is considered to be very significant. The results are expressed by mean ± standard deviation.

## 5. Conclusions

In this study, four fucoidans (F, F1, FD1 and FDS1) were extracted and purified from *Sargassum henslowianum* C.Agardh, and they were characterized by physico-chemical analysis. The four fucoidans contain l-fucose and sulfate groups. F1, FD1 and FDS1 can alleviate Adr-induced HUVEC damage and affect thrombosis-related cytokines (vWF, t-PA, PAI-1 and TF). These results suggest that fucoidan from *Sargassum henslowianum* has the potential to be developed as antithrombotic substances, but the potential structure–activity relationship and antithrombotic mechanism need to be studied in future studies.

## Figures and Tables

**Table 1:** Chemical components and Mw of fucoidans.

Fucoidan	Total Sugar (%)	Sulfate (%)	Uronic Acid (%)	Mw (Da)
F	27.97	11.45	14.47	5.677 × 10^5^
F1	35.14	16.35	12.54	4.393 × 10^5^
FD1	49.27	17.52	10.85	2.176 × 10^4^
FDS1	-	9.66	-	6.166 × 10^3^

**Table 2:** Heparin affects the levels of cytokines secreted by Adr-induced HUVEC.

vWF (U/L)	t-PA (ng/mL)	PAI-1 (μg/mL)	t-PA/PAI-1	TF (pg/mL)
1441.97 ± 4.69	20.2 ± 0.34	65.5 ± 1.02	0.31 ± 0.028	210.73 ± 12.3

## Data Availability

The data presented in this study are available from the corresponding author upon reasonable request.

## Supplementary Materials

The following supporting information can be downloaded at: https://www.mdpi.com/article/10.3390/md20050300/s1, Figure S1: Standard curves and elution curves. (A) Molecular weight standard curve (Sephacryl Smuri 300 HR), (B) Molecular weight standard curve (Superdex 75), (C) Blue glucan-2000 elution curve, (D) F elution curve, (E) F1 elution curve, (F) FD1 elution curve, (G) FDS1 elution curve.
