# Supplementary material for: Physicochemical Characterization of Fucoidans from Sargassum henslowianum C.Agardh and Their Antithrombotic Activity In Vitro
Source: Mar Drugs. 2022 Apr 28;20(5):300. doi: 10.3390/md20050300 (PMC9144781; doi:10.3390/md20050300)
Supplement: Supplementary file 1 [file marinedrugs-20-00300-s001.zip › marinedrugs-1688710-supplementary.pdf]

Fucoidans from *Sargassum henslowianum C.Agardh*: Purification, preliminary structural characterization and antithrombotic activity in vitro

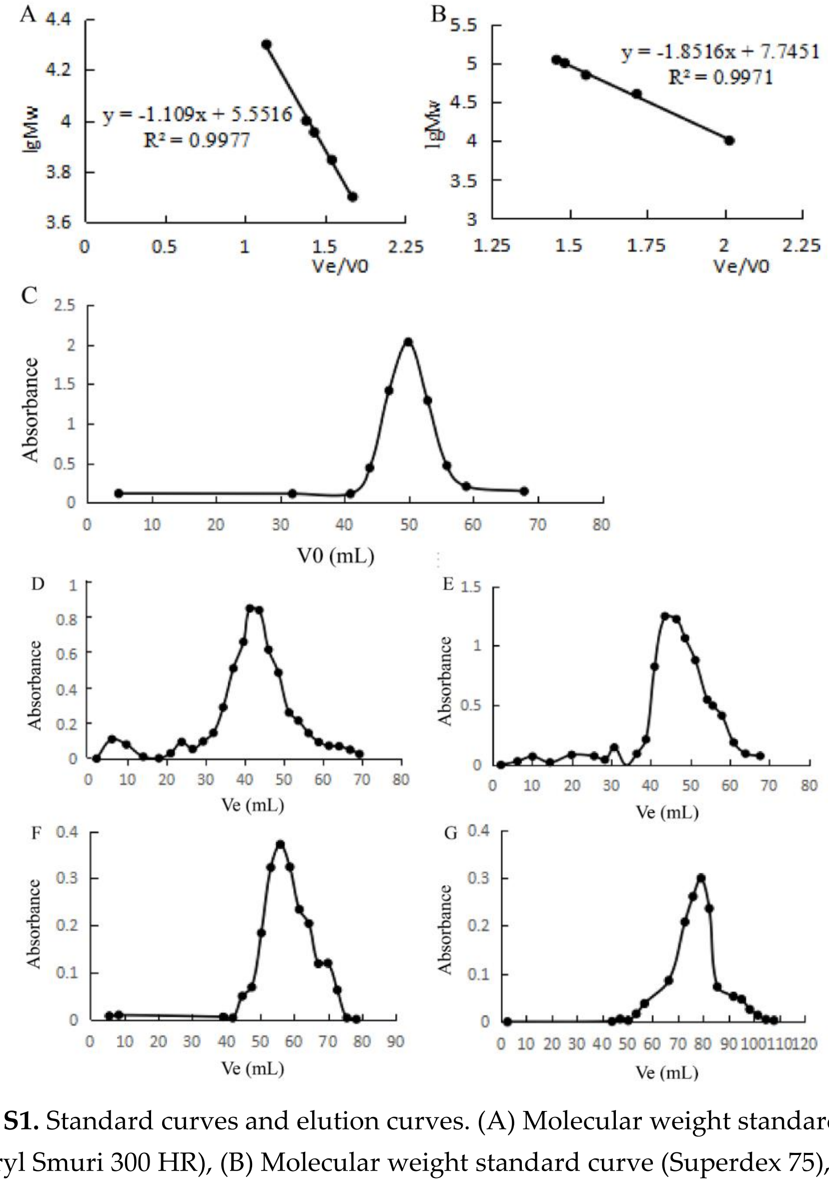

Figure S1. Standard curves and elution curves. (A) Molecular weight standard curve (Sephacryl Smuri 300 HR), (B) Molecular weight standard curve (Superdex 75), (C) Blue glucan-2000 elution curve, (D) F elution curve, (E) F1 elution curve, (F) FD1 elution curve, (G) FDS1 elution curve.
